# Detection of *EGFR* gene mutation status from pleural effusions and other body fluid specimens in patients with lung adenocarcinoma

**DOI:** 10.1111/1759-7714.13201

**Published:** 2019-10-10

**Authors:** Ping Zhang, Xiaonan Wu, Min Tang, Xin Nie, Lin Li

**Affiliations:** ^1^ Department of Oncology Beijing Hospital, National Center of Gerontology Beijing China

**Keywords:** Body fluids, circulating tumor DNA, *EGFR*, lung adenocarcinoma, next generation sequencing, plasma

## Abstract

**Background:**

Epidermal growth factor receptor (*EGFR*) gene mutation status is essential to the optimal management of lung adenocarcinoma. Liquid biopsy has advantages such as noninvasiveness, speediness, and convenience. This study aimed to detect *EGFR* gene mutations using next‐generation sequencing (NGS) from different types of body fluids from patients with lung adenocarcinoma.

**Methods:**

This was a prospective study of 20 patients with lung adenocarcinoma recruited between January 2017 and December 2018 at the Beijing Hospital. All patients had adenocarcinoma with confirmed sensitizing *EGFR* mutations. Body fluid specimens included pleural effusion, ascites, pericardial effusion, and cerebrospinal fluid. NGS was conducted to test for nine lung cancer‐related gene in body fluid supernatant free DNA, sedimentary tumor cells, and plasma free DNA.

**Results:**

The *EGFR* gene mutation abundance of body fluid supernatant free DNA was higher than that of body fluid sedimentary tumor cells and plasma free DNA specimens (100% vs. 90% vs. 80%, respectively, all *P* < 0.05). The results of *EGFR* mutation from the body fluid supernatants were consistent with the results from the tissue biopsy.

**Conclusions:**

This study showed that compared with body fluid sediment tumor cells and plasma free DNA samples, body fluid supernatant free DNA has a higher detection rate and sensitivity of tumor‐specific mutations. Free DNA obtained from body fluid supernatants could be used as high‐quality specimens for gene mutation detection in patients with lung cancer. This could be applied in treatment decisions and patient management.

## Introduction

Lung cancer is the leading cause of cancer‐related mortality worldwide.^1^ About 85%–90% of the cases are non‐small cell lung cancer (NSCLC),^2^ with adenocarcinoma representing the majority of the cases.^3^, ^4^ NSCLC mostly affects adults ≥65 years of age, tobacco smokers, and men.^2^, ^3^, ^5^ According to TNM 8, the five‐year survival varies from 92% for stage IA1 to 0% for stage IVB.^6^

A major driving gene in the development and progression of NSCLC is the epithelial growth factor receptor (*EGFR*). The two major mutations in EGFR are deletions in exon 19 (present in 45% of patients with *EGFR* mutation) and a point mutation in exon 21 (in 40% of patients with *EGFR* mutation), and both are associated with the constitutive activation of the tyrosine kinase domain.^7^, ^8^ These sensitizing *EGFR* mutations are present in 10% of Caucasian patients and 50% of Asian patients.^9^ Patients with NSCLC harboring a sensitizing *EGFR* mutation have shorter survival, higher frequency of lymph node metastasis, and poorer response to chemotherapy.^7^, ^8^ Fortunately, tyrosine kinase inhibitors (TKIs) are available and the patients with sensitizing *EGFR* mutations will respond to the TKIs,^10^ but resistance will eventually occur due to TKI‐resistant mutations in *EGFR*, or to mutations arising in other genes such as *KRAS* and *BRAF*.^3^, ^8^, ^11^, ^12^, ^13^

Therefore, determining the original *EGFR* mutation status and monitoring the changes in mutations is crucial to the management of NSCLC,^3^, ^8^, ^11^, ^12^, ^13^ but biopsies are invasive procedures and can be technically impossible in some patients. So far, the concept of liquid biopsy has expanded from blood‐based resources to urine, saliva, effusion, cerebrospinal fluid and other body fluid, which acts as a simple, fast and cost efficient alternative for monitoring of disease status, or response to treatment in multiple malignancies, including lung cancer.^14^, ^15^, ^16^, ^17^, ^18^, ^19^, ^20^ A milestone is the approval for Cobas EGFR Mutation Test v2 (cobas, Roche Diagnostic US, Indianapolis, IN, USA) by the United States Food and Drug Administration (US FDA) in 2016. Compared with traditional tissue biopsy, liquid biopsy has many advantages such as speediness and convenience, and liquid biopsy technology can be used as an effective supplement for routine tissue biopsy in clinical practice.^21^, ^22^, ^23^ Although tissue biopsy is more sensitive, the procedure of liquid biopsy is more convenient. In the past, conventional molecular pathology detection mostly used amplification refractory mutation systems (ARMS) method for *EGFR* gene detection, while sediment tumors were mostly used for body fluid‐derived specimens.^24^ These methods could only detect specific predetermined mutations and had defects in quantitative and qualitative detection. Next generation sequencing (NGS) now allows multi‐gene detection and is of clinical significance for the management of patients with NSCLC.^25^, ^26^

There is emerging evidence that important genomic information can be obtained by liquid biopsy using different body fluids, which complemented and expanded data obtained from tissue biopsies. Here, we present a study that aimed to detect *EGFR* gene mutations from different types of body fluids from patients with lung adenocarcinoma.

## Methods

### Patients

This was a prospective study of 20 patients with lung adenocarcinoma recruited between January 2017 and December 2018 at the Beijing Hospital. The study was approved by the Ethics Committee of the Beijing Hospital. All patients provided written informed consent.

All patients were diagnosed with adenocarcinoma by pathological examination. All patients were confirmed to harbor sensitizing *EGFR* mutations by the ARMS method and had available histological tumor specimens. NGS was conducted to test nine lung cancer‐related genes in body fluid supernatant free DNA, body fluid sedimentary tumor cells, and plasma free DNA. The frequencies of *EGFR* gene mutation were compared among the three specimens in each patient. The nine kinds of lung cancer‐related genes were *BRAF*, *EGFR*, *HER2*, *KRAS*, *MET*, *PIK3CA*, *ALK*, *RET*, and *ROS1*.^3^, ^8^

### Diagnostic criteria and specimens

The diagnosis of lung cancer was based on the “Guidelines for the Diagnosis and Treatment Standardization of Lung Cancer (2011 Edition)”. The patients were staged according to the International Association of Lung Cancer (IASLC) 2009 seventh edition. The pathology types were classified on the 2015 edition of the World Health Organization's Lung Cancer Histology Classification.^27^

Body fluid (either pleural effusion, ascites, pericardial effusion, or cerebrospinal fluid) (10–15 mL) and peripheral blood (10 mL in EDTA anticoagulant tubes) were collected at the same time. The body fluid samples were centrifuged to separate the cell pellet and the supernatant. NGS was performed on all three specimens (supernatant specimen, sediment cytology specimen, and plasma) to detect the nine lung cancer related genes simultaneously.

### NGS

Before lung adenocarcinoma was confirmed by pathological examination, 100–200 mL of pleural effusion and pericardial effusion, and 10 mL of cerebrospinal fluid were collected in a clean glass or plastic container. After being centrifuged at 2000 rpm for 12 minutes, cell pellet and supernatant were separately collected in the cryotube.

The blood sample was centrifuged at 1600 ***g***, 4°C for 10 minutes. After centrifugation, plasma was taken from the upper part to the 2.0 mL clean centrifuge tube. The plasma was then subjected to secondary centrifugation at 16 000 ***g***, 4°C for 10 minutes to remove all cellular contaminants and the supernatant was dispensed into new labeled 2 mL centrifuge tubes, 1.5 mL each. Besides the plasma, the red blood cells were also collected in the labeled centrifuge tube and placed immediately at −80°C.

Genomic DNA was extracted from each specimen and quantified by Qubit instruments (Life Technologies, Eugene, OR). The extraction was assessed as a failure when the total concentrations were lower than 0.1 ng/uL. DNA fragmentation was evaluated by agarose gel electrophoresis. A cSMART library was constructed and quantified by Q‐PCR. The assay detected both DNA and RNA alterations including SNV, InDel Fusion, CNV, and select gene rearrangements in *EGFR*, *ALK*, *ROS1*, *RET*, *MET*, *BRAF*, *KRAS*, *HER2*, and *PIK3CA*, full list of NGS panel was summarized in Table S1. The exons of the targeted genes were analyzed by the NextSeq CN500 platform (Hangzhou BerryGenomics Diagnostics Technology Co., Ltd., Hangzhou, CN). The data were analyzed using the Verita Trekker Enliven Genotypic Interpretation system (Berry Genomics Corporation, Beijing, CN).

### Statistical analysis

Statistical analysis was performed using SPSS 19.0 (IBM, Armonk, NY, USA. Data were presented as mean ± standard deviation (SD). Continuous variables were compared with the Student's *t*‐test. *P* < 0.05 was considered statistically significant.

## Results

### Characteristics of patients

Of the 20 patients enrolled, nine were male (45.0%) and 11 (55.0%) were female. The median age was 64 years, ranging from 38 to 85. All patients had lung adenocarcinomas with stage IV disease.

### NGS detection in different body fluids

Table 1 presents the characteristics of the tested specimens. Among body fluid supernatant free DNA, body fluid sedimentary tumor cells, and plasma free DNA samples, the tumor *EGFR* gene mutation frequency of body fluid supernatant free DNA was significantly higher than that of body fluid sedimentary tumor cells and plasma free DNA specimens. The detection rate was the lowest in plasma free DNA specimens. The sensitizing *EGFR* mutations were found in all body fluid supernatant free DNA specimens.

**Table 1:** Detection results of *EGFR* gene mutation abundance from different body fluid sources

ID	Body fluid specimen	Mutation type	Blood abundance	Body fluid supernatant abundance	Body fluid sedimentary tumor cells abundance	Metastatic sites
1	Pleural effusion	21 L858R	0.4	34.88	3.54	Pleural effusion and multiple lungs metastases
2	Pleural effusion	21 L858R	22.8	39.61	3.25	Pleural effusion, and multiple brain and bone metastases
3	Pleural effusion	19 deletion	0.27	20.03	4.28	Pleural effusion
4	Pleural effusion	21 L858R	0.2	6.78	1.11	Pleural effusion
5	Pleural effusion	19 deletion	3.72	23.06	32.56	Ascites and multiple lung metastases
6	Pleural effusion	19 deletion	1.88	27.61	40.74	Pleural effusion and multiple bone metastases
7	Pleural effusion	19 deletion	1.88	36.8	36.2	Pleural effusion and bone metastases
8	Pleural effusion	21 L858R	1.04	3.54	1.79	Pleural effusion and liver metastases
9	Pleural effusion	21 L858R	3.13	23.41	38.42	Pleural effusion and lung metastases
10	Pleural effusion	19 deletion	0.8	7.08	8.53	Pleural effusion and lung metastases
11	Pleural effusion	21 L858R	0	44.98	3.13	Pleural effusion
12	Pleural effusion	19 deletion	0.81	41.65	16.91	Pleural effusion, and peritoneum and bone metastases
13	Pleural effusion	21 L858R	0.34	1.6	2.59	Pleural effusion
14	Pleural effusion	19 deletion	0.53	4.19	2.1	Pleural effusion and lung metastases
15	Pleural effusion	19 deletion	0	40.5	5.72	Pleural effusion
16	Ascites	21 L858R	0.37	31.7	3.07	Ascites and pleural effusion
17	Pericardium	19 deletion	4.83	2.94	1.03	Multiple bone and pericardium metastases
18	Pericardium	21 L858R	0.34	0.7	0	Pericardium
19	Cerebrospinal fluid	19 deletion	0	0.21	0	Meninges
20	Cerebrospinal fluid	19 deletion	0	18.05	2.96	Brain and meninges

Two patients had negative results for sediment cells and four patients had negative results of blood tests. Two out of 15 patients with pleural effusions had negative blood tests for the sensitizing *EGFR* mutations. These two patients had no metastatic manifestation other than the pleural effusions.

In two patients with cerebrospinal fluid specimens, no mutations were detected in the blood, and the metastatic sites of these two patients were limited to the brain.

Two patients had higher abundance of *EGFR* mutation in the blood compared with that of body fluid. Further analysis revealed that these two patients had extensive systemic metastases, including multiple bone metastases and multiple lung metastases.

In addition, there were two patients with negative sediment cell detection despite repeated testing, but the free DNA detection results in the body fluid supernatant were positive.

### Mutation abundance

*EGFR g*ene mutation abundance levels varied according to the sample types. In blood samples, most patients (13/20) had *EGFR g*ene mutation abundance level less than 1% with only one over 20%, while an abundance level ranged between 1% and 5%, and over 20% was more often seen in body fluid sediment cells (11/20) and body fluid supernatant (11/20), respectively.

**Figure 1:**
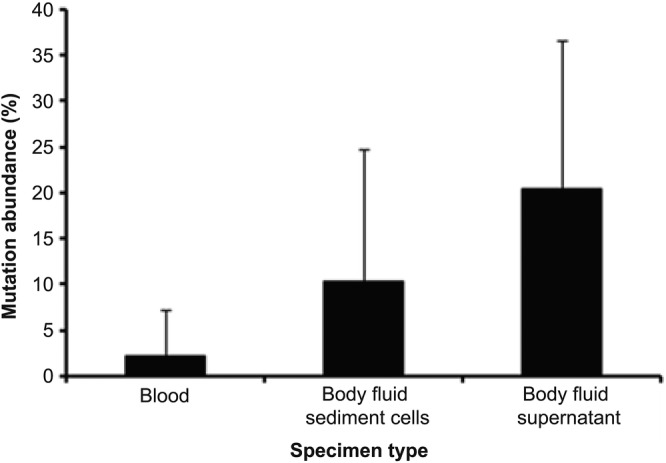
Differences of *EGFR* gene mutations abundance (%) among plasma free tumor DNA, body fluid sediment cell DNA, and body fluid free tumor DNA from patients with lung cancer.

The mean *EGFR g*ene mutation abundance was 2.17 ± 5.04%, 10.39 ± 14.18%, and 20.46 ± 16.01% in blood sample, body fluid sediment cells, and body fluid supernatant, respectively (Table 2). The abundance of *EGFR* mutations detected in free DNA of body fluid supernatant was higher than that of body fluid sediment cell DNA and plasma free DNA (*P* < 0.05) (Fig 1 and Table 2).

**Table 2:** Difference analysis of EGFR gene mutation abundance (%) in different specimen types

Specimen types	Mean ± SD	*P*‐value
Blood	2.17 ± 5.04	0.001
Body fluid sediment cells	10.39 ± 14.18*	
Body fluid supernatant	20.46 ± 16.01**,***	

* *P* < 0.05 versus blood.

** *P* < 0.05 versus blood.

*** *P* < 0.05 versus body fluid sediment cells.

## Discussion

*EGFR* gene mutation status is essential to the optimal management of lung adenocarcinoma.^3^, ^7^, ^8^ Liquid biopsy has advantages such as noninvasiveness, speediness, and convenience.^21^, ^22^, ^23^ This study aimed to detect *EGFR* gene mutations using NGS from different types of body fluids from patients with lung adenocarcinoma. The results suggest that compared with body fluid sediment tumor cells and plasma free DNA samples, body fluid supernatant free DNA has a higher detection rate and sensitivity of tumor‐specific mutations. Free DNA obtained from body fluid supernatants could be used as high‐quality specimens for gene mutation detection in patients with lung cancer. This could be applied in treatment decisions and patient management.

In the past, conventional molecular pathology detection of *EGFR* mutations mostly relied on ARMS and sediment tumors tumor cells were mainly used in the case of body fluid‐derived specimens. The ARMS platforms detect individual genes at known gene loci, with relatively fast speed and low price. On the other hand, these platforms have defects in quantitative and qualitative detection, impairing the adequate detection of *EGFR* mutations, which could lead to suboptimal patient management. NGS technology is characterized by the ability to perform multiple genetic tests on a single sample, can detect many genes, and will play an increasingly important role in guiding treatment and evaluating therapeutic effects and prognosis.^28^ In addition to tissue samples, liquid biopsy analysis of tumor materials obtained by blood or other body fluid sampling in a minimally invasive or noninvasive manner is also widely used in lung cancer diagnosis and genetic testing. Liquid biopsy has attracted much attention due to its small trauma, reproducibility, real‐time determination of treatment effects, and dynamic adjustment of treatment decisions.^21^, ^22^, ^23^ The half‐life of circulating tumor DNA is between 16 minutes and 2.5 hours,^29^ and circulating tumor DNA is considered to provide a real‐time snapshot of disease burden. In addition to blood, circulating tumor DNA was detected in various body fluids such as urine,^14^ cerebrospinal fluid,^30^, ^31^ and pleural effusion.^32^ This provides the possibility for body fluids to be used as fast and sensitive molecular detection specimens.

Among body fluid supernatant free DNA, body fluid sedimentary tumor cells, and plasma free DNA samples, we found that the tumor *EGFR* gene mutation abundance of body fluid supernatant free DNA was significantly higher than that of body fluid sedimentary tumor cells and plasma free DNA specimens. The detection rate was the lowest in plasma circulating tumor DNA. Body fluids or cytology specimens sampled near the tumor site may result in higher gene mutation abundance than the abundance found in plasma. In the present study, four patients had negative results of blood tests, while 16 (80%) had positive blood test. In a survey of several cancer types, 82% of patients with stage IV cancer have been detected with plasma circulating tumor DNA,^33^ which is similar to the present study despite two different methods being used (ARMS vs. NGS). A meta‐analysis of 27 studies published between 2007 and 2015 using a wide variety of methods and including nearly 4000 patients showed a comprehensive sensitivity of 60% in detecting plasma or serum *EGFR* mutations, with 94% specificity.^34^

In the present study, two patients had negative results for sediment cells, but all patients had positive results of supernatant‐free DNA. In low‐load diseases or certain cancer types, the concentration of circulating tumor DNA may be low, and the loss of any sampled material may reduce the sensitivity of the molecular analysis. In addition, there were two patients with negative sediment cell detection despite multiple repetitions, but the free DNA detection results in the body fluid supernatant were positive. Blood *EGFR* mutation abundance of two patients was higher than the results of body fluid test; these two patients had extensive systemic metastases, including multiple bone metastases and multiple lung metastases. Therefore, for patients with extensive systemic metastases and large tumor burden, blood free DNA detection could be considered. Indeed, the concentration of circulating tumor DNA in plasma is related to the size and stage of the tumor. A study of 640 patients with different cancer types and stages found that the median circulating tumor DNA concentration of patients with stage IV cancer was 100‐fold higher than that of stage I patients.^33^ Previous studies have also shown that the detection rate of tumor‐specific mutations in body fluid free DNA is higher than that in body fluid sedimentary tumor cells and plasma free DNA samples, and the abundance of detected mutations in body fluid free DNA is also higher than that of body fluid sedimentary tumor cells and plasma free DNA specimens,^35^, ^36^, ^37^ supporting the findings of the present study.

In this study, the mutations of two patients were not detected in both cerebrospinal fluid and blood, and the metastatic sites were only limited to the brain and meninges. Because of the high risk of brain surgery and the difficulty in obtaining brain tissue, the mechanisms of tumor metastasis to the central nervous system are difficult to study, and accurate diagnosis and treatment of brain metastases is difficult to carry out. The liquid biopsy technology could solve this dilemma based on its minimal invasiveness and high sensitivity. Because of the presence of the blood‐brain barrier, DNA released by brain tumors has a low likelihood of being detected in the plasma and cerebrospinal fluid could then be used for the detection of brain metastases and patient management.^30^, ^31^ The relationship between circulating tumor DNA levels and cancer stage suggests the prognostic utility of circulating tumor DNA in clinical practice.

The circulating tumor DNA from a liquid biopsy specimen could be from multiple tumor clones. Therefore, it could simultaneously reflect heterogeneity within a tumor^31^, ^38^, ^39^ as well as of disseminated lesions.^40^, ^41^, ^42^ Therefore, single tumor tissue biopsies may differ in mutational spectrums due to heterogeneity,^43^, ^44^ while circulating tumor DNA analysis may detect missing mutations in the corresponding tissue samples.^38^, ^40^, ^45^ Hence, detection of *EGFR* mutations in circulating tumor DNA could more comprehensively reflect the disease reality of the patients with NSCLC and guide targeted treatments. This could also provide a practical solution to avoid repeated biopsies.

The present study has limitations. Between January 2017 and December 2018, we screened 102 patients; however, during the two‐year enrollment period, only 20 were eligible with most body fluid samples being pleural effusion. We had to close the study due to the accrual limitations, which limited the sample size, as well as the types of body fluid specimens. The study should also be expanded to include patients with the entire spectrum of cancer stages and metastatic sites, and the exact metastatic sites should be correlated with the specific positive body fluids.

In conclusion, body fluid supernatant free DNA has a higher detection rate and sensitivity of tumor‐specific mutations compared with body fluid sediment tumor cells and plasma free DNA samples. Circulating tumor DNA obtained from body fluid supernatants could be used as high‐quality specimens for gene mutation detection in patients with lung cancer. This could be applied in treatment decisions and patient management.

## Disclosure

The authors have declared that no conflict of interest exists.

## Supporting information


**Table S1.** Details of NGS panel.
