# Supplementary material for: Detection of EGFR gene mutation status from pleural effusions and other body fluid specimens in patients with lung adenocarcinoma
Source: Thorac Cancer. 2019 Oct 10;10(12):2218–24. doi: 10.1111/1759-7714.13201 (PMC6885423; doi:10.1111/1759-7714.13201)
Supplement: Supplementary file 1 — Table S1. Details of NGS panel. [file TCA-10-2218-s001.docx]

Supplementary table 1 Details of NGS panel

| Targeted Areas | | Mutations | COSMIC ID* |
| --- | --- | --- | --- |
| EGFR | 18 | G719S | COSM6252 |
| EGFR | 18 | G719C | COSM6253 |
| EGFR | 18 | G719C | COSM18441 |
| EGFR | 18 | G719A | COSM6239 |
| EGFR | 19 | E746_A750del | COSM6223 |
| EGFR | 19 | E746_A750del | COSM6225 |
| EGFR | 19 | L747_A750>P | COSM12382 |
| EGFR | 19 | L747_A750>P | COSM12422 |
| EGFR | 19 | L747_T751del | COSM6254/COSM12369 |
| EGFR | 19 | E746_T751>I | COSM13551 |
| EGFR | 19 | E746_T751del | COSM12728 |
| EGFR | 19 | E746_T751>A | COSM12678 |
| EGFR | 19 | E746_S752>A | COSM12367 |
| EGFR | 19 | E746_S752>V | COSM12384 |
| EGFR | 19 | E746_S752>D | COSM6220 |
| EGFR | 19 | L747_E749del | COSM6218 |
| EGFR | 19 | L747_T751>Q | COSM12419 |
| EGFR | 19 | L747_T751>P | COSM12383 |
| EGFR | 19 | L747_T751>S | COSM6210 |
| EGFR | 19 | L747_S752del | COSM6255 |
| EGFR | 19 | L747_P753>Q | COSM12387 |
| EGFR | 19 | L747_P753>S | COSM12370 |
| EGFR | 20 | S768I | COSM6241 |
| EGFR | 20 | T790M | COSM6240 |
| EGFR | 20 | V769_D770insASV | COSM12376 |
| EGFR | 20 | D770_N771insG | COSM12378 |
| EGFR | 20 | H773_V774insH | COSM12377 |
| EGFR | 20 | C797S(1) | - |
| EGFR | 20 | C797S(2) | - |
| EGFR | 21 | L858R | COSM6224 |
| EGFR | 21 | L858R | COSM13553 |
| EGFR | 21 | L858R | COSM133630 |
| EGFR | 21 | L858R | COSM12429 |
| EGFR | 21 | L861Q | COSM6213 |
| KRAS | 2 | G12S | COSM517 |
| KRAS | 2 | G12C | COSM516 |
| KRAS | 2 | G12C | COSM513 |
| KRAS | 2 | G12R | COSM518 |
| KRAS | 2 | G12R | COSM249888 |
| KRAS | 2 | G12D | COSM521 |
| KRAS | 2 | G12D | COSM14209 |
| KRAS | 2 | G12A | COSM522 |
| KRAS | 2 | G12V | COSM520 |
| KRAS | 2 | G12V | COSM515 |
| KRAS | 2 | G13D | COSM532 |
| KRAS | 2 | G13D | COSM531 |
| KRAS | 3 | Q61K | COSM549 |
| KRAS | 3 | Q61E | COSM550 |
| KRAS | 3 | Q61R | COSM552 |
| KRAS | 3 | Q61L | COSM553 |
| KRAS | 3 | Q61P | COSM551 |
| KRAS | 3 | Q61H | COSM555 |
| KRAS | 3 | Q61H | COSM554 |
| KRAS | 4 | A146P | COSM19905 |
| KRAS | 4 | A146V | COSM19900 |
| KRAS | 4 | A146T | COSM19404 |
| BRAF | 15 | V600E | COSM476 |
| BRAF | 15 | V600E | COSM475 |
| BRAF | 15 | V600D | COSM308550 |
| BRAF | 15 | V600D | COSM477 |
| BRAF | 15 | V600K | COSM473 |
| BRAF | 15 | V600R | COSM1127 |
| BRAF | 15 | V600R | COSM1583011 |
| BRAF | 15 | V600R | COSM474 |
| ALK | 22 | T1151_L1152insT | COSM144252 |
| ALK | 22 | L1152R | COSM97185 |
| ALK | 22 | C1156Y | COSM99136 |
| ALK | 22 | I1171N | COSM28498 |
| ALK | 23 | F1174L | COSM28057 |
| ALK | 23 | L1196M | COSM99137 |
| ALK | 23 | G1202R | COSM144250 |
| ALK | 23 | D1203N | - |
| ALK | 23 | S1206Y | COSM144251 |
| ALK | 25 | G1269A | COSM1169707 |
| ALK |  | Fusion | - |
| HER2 | 20 | A775_G776insYVMA | COSM21599/ COSM20959 |
| HER2 | 20 | A775_G776insYVMA | COSM12558/ COSM133608 |
| HER2 | 20 | G776>VC | COSM12552 |
| HER2 | 20 | G776>VC | COSM12553 |
| HER2 | 20 | G776>VC | COSM85995 |
| HER2 | 20 | P780_Y781insGSP | COSM12555 |
| HER2 | 20 | P780_Y781insGSP | COSM12556 |
| HER2 | 20 | P780_Y781insGSP | COSM303948 |
| PIK3CA | 2 | R88Q | COSM746 |
| PIK3CA | 10 | E542K | COSM760 |
| PIK3CA | 10 | E545K | COSM763 |
| PIK3CA | 10 | E545D | COSM27374 |
| PIK3CA | 10 | E545D | COSM765 |
| PIK3CA | 21 | H1047R | COSM775 |
| PIK3CA | 21 | H1047L | COSM776 |
| MET |  | Exon14_skipping | - |
| RET |  | Fusion | - |
| ROS1 |  | Fusion | - |
